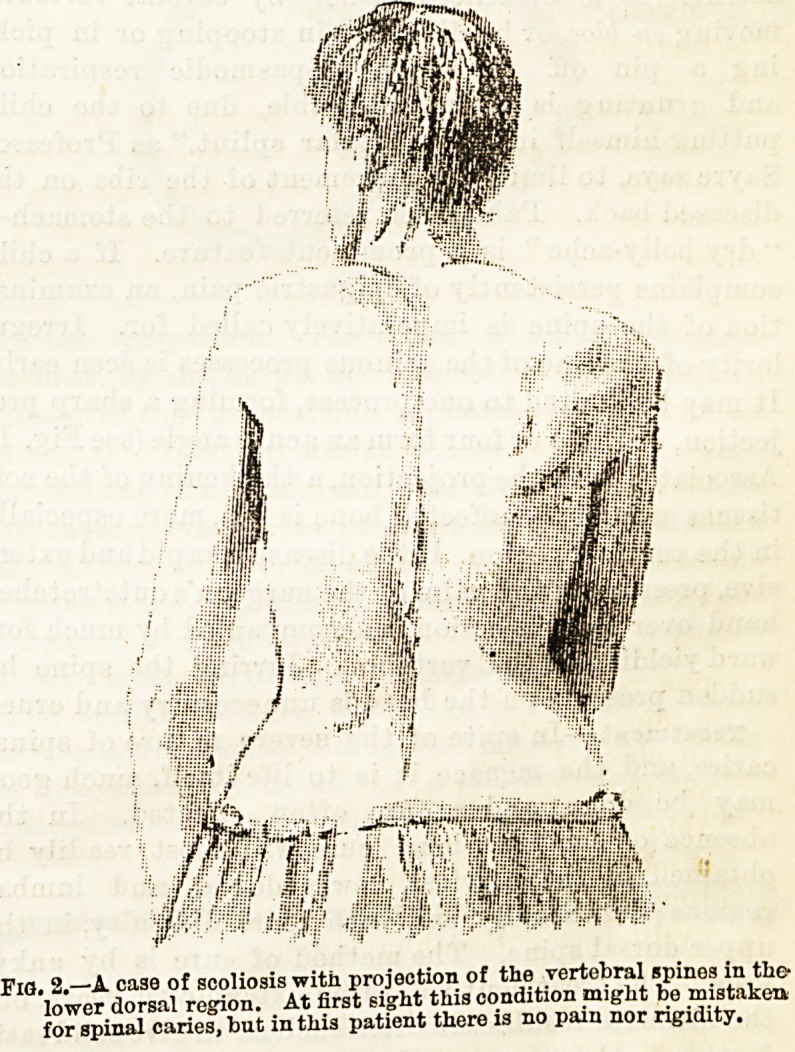# The Treatment of Angular Deformity of the Spine, or Spinal Caries

**Published:** 1894-07-14

**Authors:** A. H. Tubby

**Affiliations:** Surgeon to the National Orthopædic Hospital, and Surgeon to Out-patients, Evelina Hospital for Sick Children, &c.


### Medical Progress and Hospital Clinics.

[*The Editor will be glad to receive offers of co-operation and contributions from members of the profession. All letters should be addressed to* The Editor, The Lodge, Porchester Square, London, W.]

### THE TREATMENT OF ANGULAR DEFORMITY OF THE SPINE, OR SPINAL CARIES.

By A. H. Tubby, M.S., M.B.Lond., F.R.C.S.Eng.

Surgeon to the National Orthopædic Hospital, and Surgeon to Out-patients, Evelina Hospital for Sick Children, &c.

In dealing with this difficult and somewhat complicated subject it is necessary to make a few general remarks on the disease itself, which must be borne in mind in considering the various points of treatment.

Fig 1.—A case of angular deformity of the spine, with projection in the upper dorsal region.

Spinal caries is a disease of the young. It is almost unknown after forty years of age, unless due to the pressure of aneurism, syphilis, or growth. Heredity plays a large part in its production, and many cases are tubercular from the first. But I am far from admitting that all cases are tainted by tuberculosis. On the contrary; for it does happen that a healthy child of sound parentage, injured in the back suffers from caries of a simple nature, just as healthy young male adults get acute periostitis, or simple ostitis following a kick on the shin. The importance of recognising the tubercular taint lies in the gravity of the prognosis, and the greater care and longer duration of treatment. As to the region affected; all authors agree that it is most frequent in the dorsal region, due it may be to the fact that there are more dorsal than cervical or lumbar vertebræ. When disease occurs in the mid-region of the back it runs a longer course than elsewhere, because of the constant movement of the ribs in respiration, and the difficulty of keeping the part at rest. Deformity, or backward projection, is more likely to occur, as the normal physiological curve has its convexity backwards in this region. The immediate effect on the weakened dorsal spine of the weight of the arms and shoulders in increasing any slight initial deformity must be borne in mind.

Important, too, in considering the question of treatment is the part of the vertebræ affected. In nearly all cases it is the bodies, due to their being largely composed of cancellous bone, and mainly supporting the body weight and bearing all the sudden shocks and jars to which the spine is liable. The great depth of the bodies from the surface bears a close relation to the persistence of the discharge of some spinal abscesses. In many tubercular cases, it frequently happens that there are several foci of disease in the spine; these break down and give rise either to very severe deformity, or cause prolonged suppuration, which cannot be stopped owing to the size and inaccessibility of the sequestra.

What are the natural methods of cure? In non-tubercular cases we may take it that the traumatic ostitis rarely results in extensive caries. It is precisely in these cases that dry caries follows, so that no suppuration ensues: The granulation-tissue frequently ossifies, and the result is a certain amount of limited bony ankylosis. In tubercular cases, however, a large amount of granulation tissue forms, and a considerable portion of bone is excavated. As the destruction of the bodies proceeds, the opposed surfaces of granulation tissue come together, and with the extrusion of all diseased bone, coalesce, and finally ossify; so that a bridge of new bone is formed across the gap. But the destruction is often out of all proportion to the repair, and hence much deformity results. Nor is the diseased bone always quickly got rid of; in some patients it is not at

Fig. 2.—A case of scoliosis with projection of the vertebral spines in the lower dorsal region. At first sight this condition might be mistaken for spinal caries, but in this patient there is no pain nor rigidity.

all, and then the case ends badly: it sets up suppuration by the irritation of its presence, and much of the granulation tissue wastes away as pus, carrying with it particles of bone either microscopic in size or as small sequestra. Therefore caries of the spine is so frequently aggravated by severe complications, such as abscess, severe deformity altering the shape of the back and chest, paralysis, and lesions of the heart and lungs, owing to their displacement and compression. But these must be dealt with in separate articles.

**The Symptoms of Spinal Caries.**—By careful attention to these, we know approximately the extent and stage of the disease in an individual case. The history of the trouble should always be received with caution. Only, when the parent, presuming we are dealing with a child, can give a very decided and dogmatic account of a severe fall occurring on one particular occasion, and in the absence of tuberculosis either in parents or child, are we justified in regarding the case as purely traumatic. All vague accounts are to be regarded with suspicion. But, at the same time, the careful surgeon will not neglect to inquire into the child's antecedents, so as to be able to give a reliable opinion as to the future progress of the case. The attitude and gait are characteristic. The child is unable to stand for more than a few moments without support. If the disease is cervical, the carriage is military and erect; if in the dorsal or lumbar region, the patient leans forward as he walks, frequently resting the hands on the thighs, a method of progression indicative of psoas contraction. In some cases the gait is sidling. The general appearance is that of ill-health, and the expression pained and anxious.

The child should now be stripped, and muscular rigidity be looked for. This is a most valuable sign, and is present from the first, although often of limited extent. It is evidenced either by several vertebræ moving *en bloc*, or by difficulty in stooping or in picking a pin off the floor. Spasmodic respiration and grunting is often noticeable, due to the child putting himself in a "muscular splint," as Professor Sayre says, to limit the movement of the ribs on the diseased back. Pain, often referred to the stomach—"dry belly-ache" is a prominent feature. If a child complains persistently of epigastric pain, an examination of the spine is imperatively called for. Irregularity of the line of the spinous processes is seen early. It may be limited to one process, forming a sharp projection, or three or four form an acute angle (see Fig. 1). Associated with the projection, a thickening of the soft tissues around the affected bone is felt, more especially in the cervical region. If the disease is rapid and extensive, pressure of the palm of the surgeon's outstretched hand over the projection is accompanied by much forward yielding of the vertebræ. Jarring the spine by sudden pressure on the head is unnecessary and cruel.

**Treatment.**—In spite of the severe nature of spinal caries, and the menace it is to life itself, much good may be done, and a cure often effected. In the absence of complications, cure will most readily be obtained in the cervical, lower dorsal, and lumbar regions; less readily, and with more difficulty, in the upper dorsal spine. The method of cure is by ankylosis. The treatment will often extend over years, but the ultimate results are often such as to give solid satisfaction to the surgeon. Treatment may be described as general and local. The former includes all-round hygienic measures, and especially fresh air and sunshine. The food should be easily digestible and plentiful. Cod-liver oil, cream, milk, or one of the malt extracts, are useful; and iron, in the form of syr. ferri. phosphates Co., or syr. ferri iodidi ʒj. twice daily is useful.

**Local Treatment.**—The indications are four in number: to fix the vertebral column, and place it in the best possible conditions for healing; to remove the weight of the upper part of the body from the diseased vertebræ; to prevent unnecessary deformity by supporting the trunk in front; and if deformity has occurred to limit its increase. To carry out these indications we have two means at our disposal—recumbency, and the use of retentive apparatus.

Recumbency—when necessary: (1) In all acute cases, where there is much pain, distress, and impairment of the general health; (2) when, on employing the palm-pressure test, mentioned above, to the back, it is found very yielding; (3) where paralysis and abscess are threatened; (4) in cases of cervical and lower lumbar caries; (5) in such patients as become easily tired on their feet, and in those who, apparently well supported mechanically, desire frequently to lie down. The immediate effects are good. The pain disappears, the nervous irritability is lost, the face loses its drawn and anxious expression, and the patient often puts on fat, although the muscles sensibly decrease in size. The duration of recumbency can scarcely be specified in set terms of months. Each individual case must be judged on its merits. Suffice it to say that when the back is felt to be consolidated, and the child can be sat up carefully for a few minutes with a little aid from the nurse, or when he attempts to turn over, then is the time for supporting apparatus. While the patient is lying supine, some slight means of restraint, such as a woollen band across the chest is often necessary. It acts as an efficient physical restraint in young children, and in older as a moral one. Extension, too, must be added when the cervical spine is affected. In the next stage of treatment a combination of partial recumbency and fixative appliances is useful, thereby allowing some exercise of the limbs and more fresh air.

**Fixative and Supporting Apparatus—Tests of Efficiency.**—1. All complicated machines should be avoided. 2. Appliances should be so adjusted as to fix firmly the site of disease and transfer pressure from diseased to healthy spots. 3. They should be comfortable and cleanly. 4. Pressure on and chafing of the skin are to be avoided. 5. They should be such as can be readily applied by the surgeon himself. In the plaster of Paris and poroplastic felt jackets nearly all these requirements are fulfilled.

In applying the plaster of Paris jacket, the following points should be attended to. There are three fixation points to be made: the pelvis below the crests of the ilia, the front of the chest above, and the site of disease behind. Therefore, let the pelvic band be carried well down to the trochanters, and make the jacket reach up well in front, and place extra rolls of bandage over the three fixation points. Pads of cotton wool around prominent bones are necessary, and do not forget the use of the "dinner-pad." Poroplastic jackets have the advantages over the plaster of Paris that they are more closely fitting to the figure; they are easily removable, and allow of greater personal cleanliness; they set more rapidly, and are more durable, lasting with care twelve to eighteen months. Their chief disadvantage is that they are not so supporting. Over the mammæ, upper part of the chest and crests of the ilia, and lower part of the abdomen they are necessarily left unstiffened, and hence allow the body to "settle" somewhat. These difficulties are to a certain extent overcome by strengthening them posteriorly with stout steel bands.

When may treatment be dispensed with in spinal caries? 1. The absence of pain is no test, since this occurs naturally, if a support be worn; but if pain ensue on the removal of the jacket, it must be again resorted to. 2. When the spine is firmly fixed, and the deformity has remained stationary for some time. 3. If a recession of the deformity has been gained and maintained some months. 4. If the general health shows no signs of retrogression, 5. Treatment must be continued longer in tubercular cases.

The chief complications of angular deformity, viz., abscess and paralysis, will form the subject of a future article.

## Figures and Tables

**Fig 1. f1:**
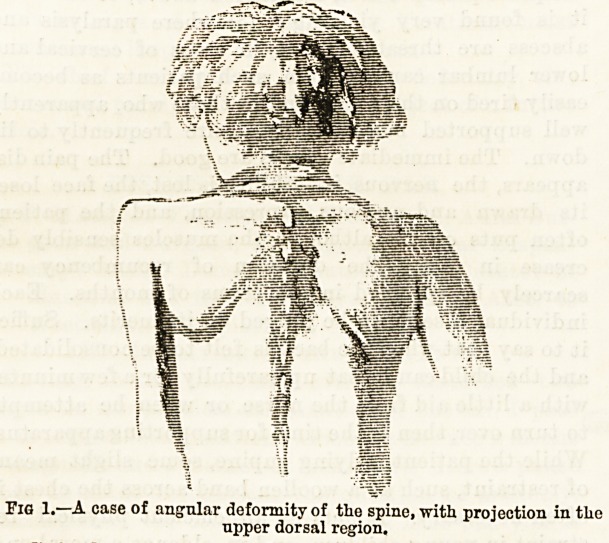


**Fig. 2. f2:**